# Metformin’s effect on metabolic dysfunction-associated steatotic liver disease through the miR-200a-5p and AMPK/SERCA2b pathway

**DOI:** 10.3389/fphar.2024.1477212

**Published:** 2024-12-17

**Authors:** Hang Chen, Minshan Huang, Dan Zhang, Hui Wang, Da Wang, Mengwei Li, Xianmei Wang, Rui Zhu, Jianjun Liu, Lanqing Ma

**Affiliations:** ^1^ The First Affiliated Hospital, Yunnan Institute of Digestive Disease, Kunming Medical University, Kunming, China; ^2^ State Key Laboratory for Conservation and Utilization of Bio-Resources in Yunnan, School of Life Sciences, Yunnan University, Kunming, China; ^3^ Yunnan Key Laboratory of Breast Cancer Precision Medicine, Academy of Biomedical Engineering, Kunming Medical University, Kunming, China

**Keywords:** MASLD, metformin, miR-200a-5p, AMPK, SERCA2b

## Abstract

**Introduction:**

Metformin has shown benefits in treating metabolic dysfunction-associated steatotic liver disease (MASLD), but its mechanisms remain unclear. This study investigates miR-200a-5p’s role in the AMPK/SERCA2b pathway to reduce liver fat accumulation and ER stress in MASLD.

**Methods:**

A PA cell model induced by palmitic and oleic acids (2:1) was used to assess lipid accumulation via Oil Red O and Nile Red staining. mRNA levels of miR-200a-5p and lipid metabolism genes were measured with RT-PCR, and AMPK, p-AMPK, and SERCA2b protein levels were analyzed by Western blotting. The interaction between miR-200a-5p and AMPK was studied using a luciferase reporter assay. A high-fat diet-induced MASLD mouse model was used to evaluate metformin’s effects on liver steatosis and lipid profiles. Serum miR-200a-5p levels were also analyzed in MASLD patients.

**Results:**

In the PA cell model, elevated miR-200a-5p and lipid metabolism gene mRNA levels were observed, with decreased AMPK and SERCA2b protein levels. miR-200a-5p mimic reduced AMPK and SERCA2b expression. Metformin treatment reduced liver steatosis and lipid deposition in mice, normalizing miR-200a-5p, lipid metabolism gene mRNA, and AMPK/SERCA2b protein levels. Elevated serum miR-200a-5p was detected in MASLD patients.

**Discussion:**

These findings suggest that metformin alleviates lipid deposition and ER stress in MASLD through the modulation of the AMPK/SERCA2b pathway via miR-200a-5p.

## 1 Introduction

Non-Alcoholic Fatty Liver Disease (NAFLD) is characterized by liver fat accumulation without excessive alcohol consumption, affecting approximately 25% of the global population (Abdelmalek, 2021; Stols-Goncalves et al., 2019). Recently, it was proposed to be renamed “steatotic liver disease” (SLD) (Rinella et al., 2024), which includes various classifications: metabolic dysfunction-associated SLD (MASLD), MASLD with increased alcohol intake, alcohol-related liver disease, SLD with specific etiologies, and cryptogenic SLD. MASLD is characterized by hepatic steatosis along with at least one of five cardiometabolic risk factors of metabolic syndrome (Rinella et al., 2024; Rinella and Sookoian, 2024). MASLD ranges from simple steatosis to severe forms leading to fibrosis, cirrhosis, and hepatocellular carcinoma (Cotter and Rinella, 2020; Benedict and Zhang, 2017; Asgharpour et al., 2016). It is associated with metabolic complications such as insulin resistance, endoplasmic reticulum (ER) stress, dyslipidemia, and cardiovascular diseases (Friedman et al., 2018). Projections indicate MASLD may become the leading cause of liver transplants by 2030 due to rising living standards (Mundi et al., 2020; Huang et al., 2021; Wang et al., 2020; Barbier-Torres et al., 2020). Given its prevalence and severe health impacts, research on MASLD prevention and management is crucial.

Metformin, widely used for type 2 diabetes mellitus (T2DM), shows promise in ameliorating MASLD. It works through multiple pathways: inhibiting mitochondrial respiratory chain complex I, increasing AMP ratio to activate AMPK, reducing hepatic gluconeogenesis, and improving peripheral glucose uptake (Fullerton et al., 2013; El-Mir et al., 2000). Metformin also lowers serum non-esterified fatty acids, inhibits lipogenic proteins, promotes acetyl-CoA carboxylase phosphorylation, and upregulates carnitine palmitoyltransferase-1 expression (Pinyopornpanish et al., 2021; Conde de la Rosa et al., 2015). Additionally, metformin alleviates ER stress and reduces lipid peroxidation, providing anti-inflammatory and antifibrotic effects (Lee et al., 2020; Yogalakshmi et al., 2019; Kim et al., 2019). Sarcoplasmic Reticulum Calcium-Transporting ATPases (SERCA) translocate Ca2^+^ into the ER lumen, and targeting the AMPK/SERCA2b pathway may reduce ER stress (Park et al., 2010; Jung et al., 2018).

MicroRNAs (miRNAs), approximately 22 nucleotides long, are non-coding RNAs that regulate gene expression by targeting mRNA. Recent studies, including one by Ezaz et al., have explored the association between circulating miRNAs (such as miR-34a, miR-122, miR-191, miR-192, and miR-200a) and histopathological changes in patients with MASLD, confirmed through liver biopsies. Significant upregulation of miR-34a, miR-122, miR-192, and miR-200a was observed (Ezaz et al., 2020). Notably, miR-200a expression was closely linked with MASLD severity. Furthermore, studies have shown that metformin can enhance miR-200 family expression (Wang et al., 2018). However, the role of miR-200a, particularly its interaction with the AMPK pathway in the context of metformin’s therapeutic action against MASLD, remains unclear.

In this study, we demonstrated that metformin treatment significantly ameliorates hepatic lipid accumulation in high-fat diet (HFD) group. The protective effect of metformin may be intricately linked with the regulation of miR-200a-5p. Our observations suggest that metformin might exert its therapeutic benefits in MASLD through miR-200a-5p modulation, regulating the AMPK/SERCA2b pathway. This mechanism could lead to improved lipid deposition and reduced ER stress in MASLD. These findings offer fresh insights into metformin’s potential as a therapeutic agent for MASLD.

## 2 Methods

### 2.1 Ethics statement

The experiments were performed in compliance with the European Communities Council Directive dated 24 November 1986 (86/609/EEC). This research received approval from the Animal Care and Use Committee at Kunming Medical University (project license number: kmmu20211520). Prior to the commencement of the sample collection process, we obtained full ethical clearance from the Ethics Committee of the First Affiliated Hospital of Kunming Medical University (project license number: L-170/2022).

### 2.2 Animals

The experimental protocols and animal handling in this study were conducted in strict accordance with the Guide for the Care and Use of Laboratory Animals as published by the China National Institutes of Health. Adult C57BL/6J mice (male, 21–23 g), aged 6 weeks, were procured from the Nanjing Biomedical Research Institute of Nanjing University, under license number SCXK [S] 2005-0019. The mice were accommodated in standard laboratory cages, with unrestricted access to food and water. The environmental conditions were maintained at a temperature of 20°C ± 2°C and a relative humidity of 50% ± 5%. The mice were randomly allocated into three groups: a Control group, maintained on a regular diet; a high-fat diet (HFD) group, where mice were fed a high-fat diet for 14 weeks and received daily oral gavages of sterile saline during the final 9 weeks; and an HFD + Metformin (Met) group, in which HFD-fed mice were administered daily oral gavages of metformin (300 mg/kg) for the last 9 weeks.

### 2.3 Cell lines and culture

All cell lines used in this study were provided by the Stem Cell Bank of the Chinese Academy of Sciences, with the AML12 cell line cataloged under SCSP-550. The AML12 cells were maintained in a complete medium (catalog number CM-0602) supplied by Procell Life Science and Technology Co., Ltd., China. Culturing conditions were set in a humidified incubator at 37°C with an atmosphere containing 5% CO2. Palmitic acid (PA, catalog number P9767-5, Sigma) and oleic acid (OA, catalog number O7501, Sigma) were prepared as 1 mM stock solutions. Working solution was formulated by mixing OA and PA solutions in a 2:1 ratio. This working solution was then added to the cultured cells for 24 h.

### 2.4 Clinical sample

We collected clinical samples comprising peripheral blood serum from two distinct groups: 10 healthy volunteers and 10 patients diagnosed with MASLD. To ensure a comprehensive understanding of the health status of each participant, we gathered detailed demographic information and conducted biochemical tests for each individual’s sample.

### 2.5 Bioinformatics analysis

The raw data for the GSE94799 dataset was downloaded from the GEO database (https://www.ncbi.nlm.nih.gov/geo/). We conducted an analysis to compare gene expression levels across various samples, specifically between Control and HFD groups, as well as between HFD and HFD + Met groups. Genes that exhibited an increase in baseline expression by more than 1.75-fold in the HFD group and a decrease of more than 1.75-fold in the HFD + Met group were identified as differentially expressed. The data visualization in this study was performed using the R programming language (Version 4.3.0), along with the “ComplexHeatmap” (Version 2.16.0) and “ggplot2” (Version 3.4.2) packages.

### 2.6 Histology

Liver tissues were processed by embedding in OCT compound and then rapidly frozen in liquid nitrogen. These tissues were then sectioned into 5 μm slices. For evaluating lipid accumulation, the sections were stained using an Oil red O solution for 10 min at 60°C. After the staining process, the sections were washed with 60% isopropyl alcohol and subsequently counterstained with hematoxylin to accentuate the nuclei. In addition, liver sections underwent hematoxylin and eosin (H&E) staining for further examination. Image analysis was performed using ImageJ software.

### 2.7 Nile red staining

Cells from each group were cultured in confocal dishes under optimal conditions. A working solution was prepared by diluting 1 mg/mL Nile red (catalog number D8371, Solarbio) in PBS at a 1:500 ratio. This diluted solution was then dispensed onto the cell slides, which were incubated at 37°C for 30 min to facilitate staining. Following a rinse with PBS, the slides were examined using a confocal microscope, and the fluorescence intensity was quantitatively determined.

### 2.8 Western blotting

Fresh liver tissues were pulverized under liquid nitrogen, and high-efficiency RIPA lysate (catalog number 89900, Thermo) was utilized for protein extraction. The resultant protein samples were then loaded (20 μg/10 μL per well) and resolved via sodium dodecyl sulfate-polyacrylamide gel electrophoresis (SDS-PAGE) on gels comprised of either 10% or 12.5% acrylamide. Subsequently, the separated proteins were transferred onto polyvinylidene fluoride (PVDF) membranes (catalog number ISEQ00010, Millipore). The membranes were then blocked with 5% skim milk in TBST for 2 h at room temperature. Following blocking, the membranes were incubated with primary antibodies targeted against AMPK (1:1000), phosphorylated AMPK (p-AMPK, 1:1000), SERCA2b (1:1000), GAPDH (1:1000), and Actin (1:1000). After thorough washing and incubation with appropriate secondary antibodies, the blots were visualized using a ChemiDoc XRS imaging system. The quantification of target proteins was achieved by normalizing their relative intensities in the experimental groups to those in the control group.

### 2.9 Quantitative real-time PCR (qRT-PCR)

Total RNA was extracted using the Total RNA Rapid Extraction Kit (catalog number YFXM0011P, Yifeixue). cDNA synthesis was then carried out using the RT First Strand cDNA Synthesis Kit (catalog number YFXM0012, Yifeixue). Quantitative real-time PCR (qRT-PCR) assays were performed with the SYBR (catalog number YFXM0006, Yifeixue) on a Bio-Rad CFX96 Touch system. The specific primers employed for the qRT-PCR are detailed in Supplementary Table S1. All qRT-PCR experiments were conducted in triplicate. GAPDH was utilized as the normalization control. Gene expression analysis was conducted using the ^ΔΔ^Ct method.

### 2.10 Dual-luciferase reporter assays

For the dual-luciferase reporter assay, constructs of AMPK-WT and AMPK-Mut were prepared and transfected into HEK293 cells along with either the control mimic (NC) or the miR-200a-5p mimic. Following transfection, the luciferase activities were quantified using a Dual-Luciferase Reporter Assay System (catalog number E2920, Promega). Microplate Luminometer was employed to microplate assays.

### 2.11 Statistical analyses

Statistical evaluations in this study were conducted utilizing GraphPad Prism version 9. The data are presented as the mean ± standard error of the mean (SEM). The statistical significance of the differences was assessed using one-way ANOVA, followed by a Student-Newman-Keuls test. Differences were deemed statistically significant at a threshold of *p* < 0.05.

## 3 Results

### 3.1 Integrated analysis of miRNA regulation in AMPK pathway under high-fat diet and metformin treatment

**FIGURE 1:**
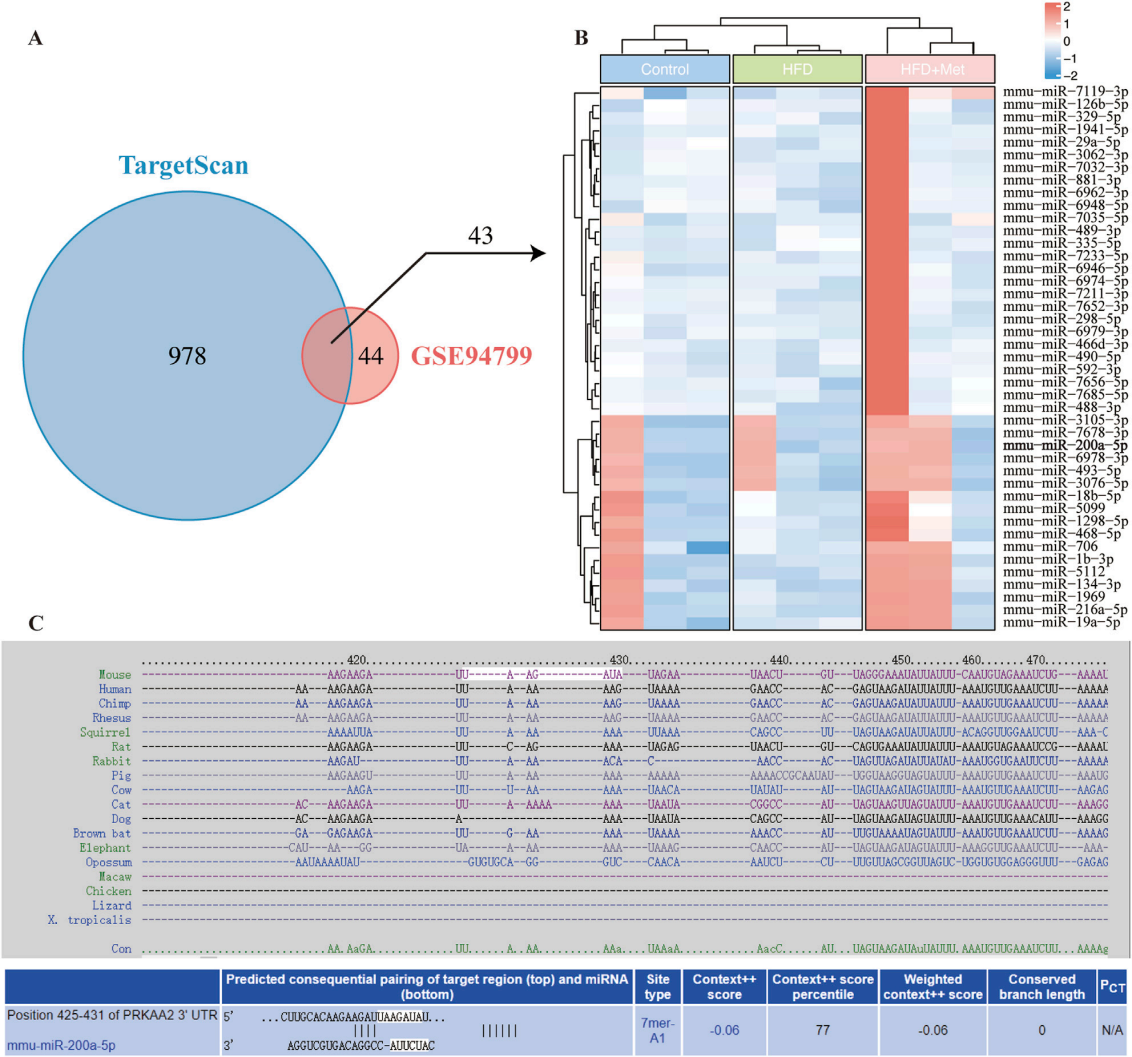
Integrative Analysis of miRNA-AMPK Interactions and Differential miRNA Expression in Response to Metformin Treatment in GSE94799. **(A)** Venn diagram displays miRNAs in TargetScan and differentially miRNA expression in GSE94799. **(B)** The heatmap showed the differential miRNAs in GSE94799. **(C)** Complementarity between AMPK gene and miRNA-200a-5p.

To investigate miRNA regulation in the AMPK pathway under high-fat diet (HFD) and metformin treatment, we used TargetScan to predict miRNAs binding to AMPK, identifying 1,021 candidates. Using the GSE94799 dataset, we filtered miRNAs that increased in the HFD group and decreased by more than 1.75-fold in the HFD + Met group, resulting in 87 miRNAs. Venn diagram analysis identified 43 miRNAs meeting these criteria (Figure 1A). A heatmap visualized the expression patterns of these miRNAs (Figure 1B), and TargetScan data illustrated potential binding sites between PRKAA2 (AMPK) and miR-200a-5p (Figure 1C).

### 3.2 Metformin alleviates obesity, hepatic steatosis, and lipid metabolism in HFD mice

**FIGURE 2:**
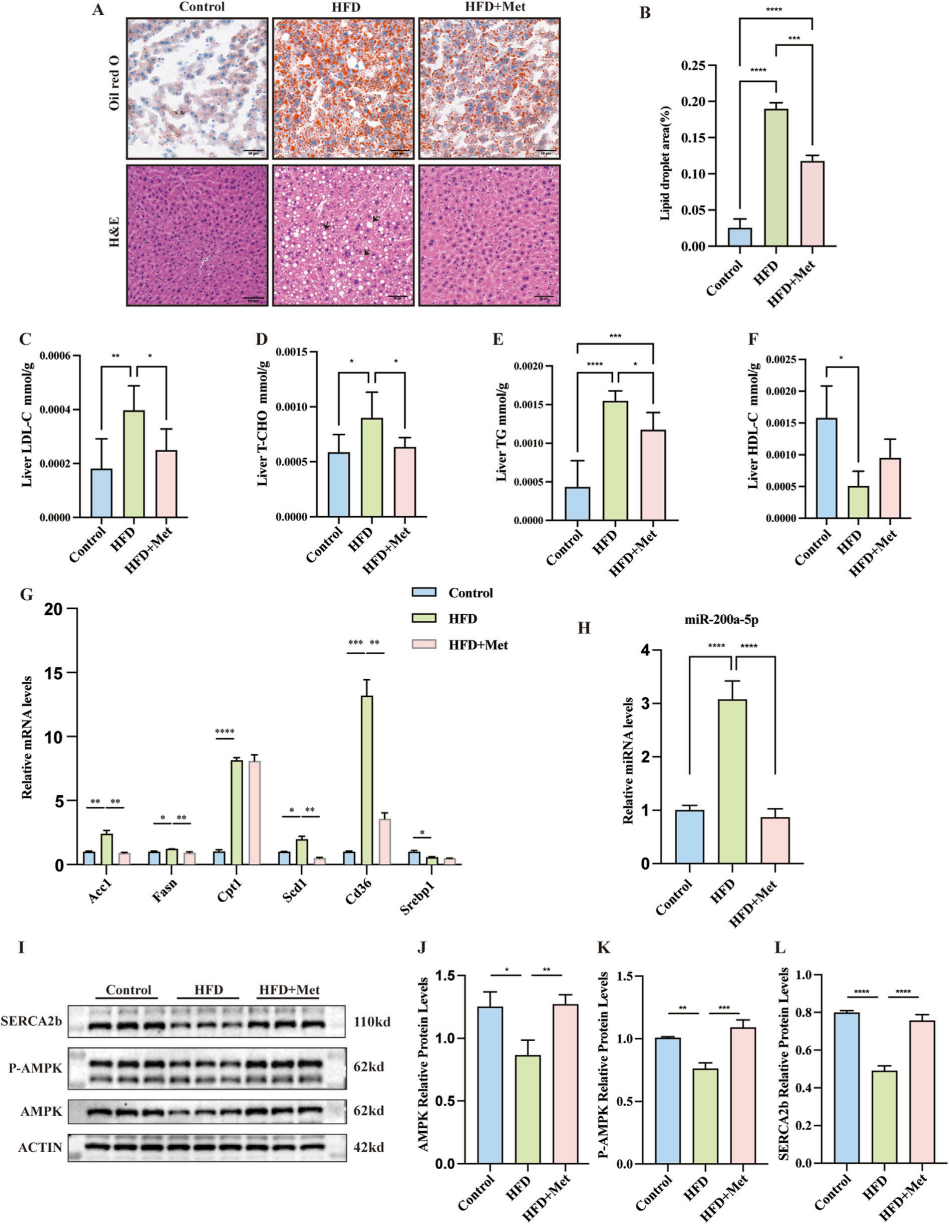
Effects of Metformin on Hepatic Steatosis and Lipid Metabolism in Mice Fed a High-Fat Diet (HFD). **(A, B)** Representative Oil Red O and Hematoxylin and Eosin (H&E) stained sections of liver tissues from each of the three study groups (×200 magnification; scale bar: 50 μm). Arrows highlight the presence of fat vacuoles or lipid droplets. **(C–F)** Levels of Low-Density Lipoprotein Cholesterol (LDL-C), Total Cholesterol (TC), Triglycerides (TG), and High-Density Lipoprotein Cholesterol (HDL-C) measured in the three groups, with n = 6 per group. **(G)** Quantitative Real-Time PCR analysis revealing mRNA expression of lipid metabolism-related genes in liver tissues. **(H)** Quantitative Real-Time PCR analysis showing miRNA-200a-5p expression in liver tissues. **(I–L)** Protein expression of AMPK, phosphorylated AMPK (p-AMPK) and SERCA2b, as detected by Western blot, was normalized to actin. Data were analyzed using one-way ANOVA. All values are expressed as mean ± SEM, with n = 3 per group. The groups are as follows: Control (normal diet); HFD (high-fat diet); HFD + Met (mice on a high-fat diet receiving oral gavage of metformin at 300 mg/kg daily for the last 9 weeks). Comparisons were made between the HFD group and the Control group, as well as between the HFD + Met group and the HFD group. Significance levels are indicated as follows: *p < 0.05, **p < 0.01, ***p < 0.001, ****p < 0.0001.

To assess metformin’s effect on MASLD, HFD mice received daily oral doses of sterile saline or metformin for 9 weeks. Hematoxylin and Eosin (H&E) and Oil Red O staining showed reduced hepatic lipid accumulation in the HFD + Met group (Figures 2A, B). Metformin improved hepatic LDL-C, TC, TG, and HDL-C levels (Figures 2C–F). We also analyzed the expression levels of lipid metabolism-related genes in the liver tissues, including Acc1, Fasn, Cpt1, Scd1, Cd36, and Serbp1 mRNA. Notably, the expression of Fasn, Acc1, Scd1, and Cd36 were found to be elevated in the HFD group but decreased in the HFD + Met group (Figure 2G).

### 3.3 Modulation of miR-200a-5p and AMPK pathway in response to high-fat diet and metformin treatment

In our study, we observed distinct changes in the expression of miR-200a-5p and key components of the AMPK pathway in both the HFD group and the HFD + Met group. Specifically, miR-200a-5p levels were elevated in the HFD group but showed a decrease in the HFD + Met group (Figure 2H). Conversely, the expression of AMPK and p-AMPK was reduced in the HFD group but increased in the HFD + Met group. We assessed the expression of SERCA2b, SERCA2b levels were decreased in the HFD group but restored in the HFD + Met group (Figures 2I–L; Supplementary Figure S1).

### 3.4 Lipid metabolism in the PA cell model

**FIGURE 3:**
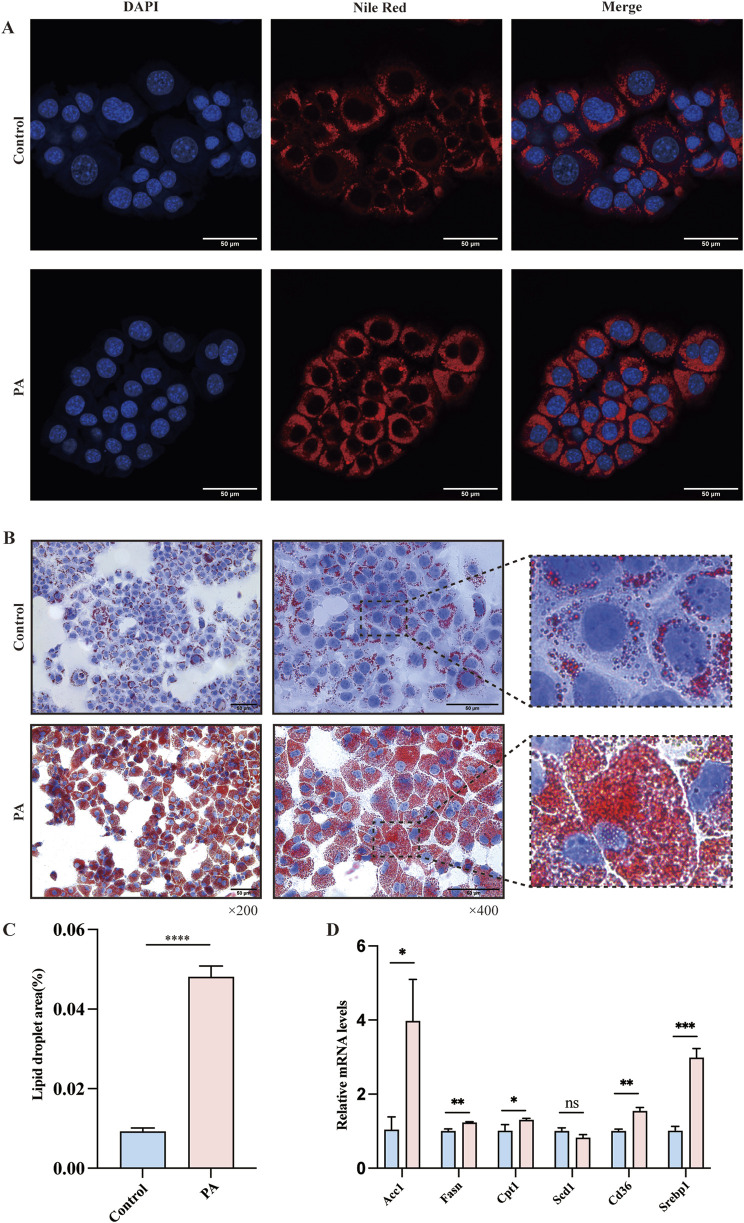
Evaluation of Lipid Metabolism in AML12 Cells via Nile Red and Oil Red O Staining. **(A)** Nile Red staining confirmed the Lipid Metabolism of AMl12 cells in different groups. **(B, C)** Oil Red O staining confirmed the Lipid Metabolism of AMl12 cells in different groups. **(D)** Quantitative Real-Time PCR analysis revealing mRNA expression of lipid metabolism-related genes in AML12 Cells. Data were analyzed using one-way ANOVA. All values are expressed as mean ± SEM, with n = 3 per group. The groups are as follows: Control (normal group); PA (A mixture of Oleic and Palmitic Acids in a 2:1 ratio group). Comparisons were made between the PA group and the Control group. Significance levels are indicated as follows: *p < 0.05, **p < 0.01, ***p < 0.001, ****p < 0.0001.

To further explore the interaction between miR-200a-5p and AMPK within the context of hepatic lipid accumulation, we established a cellular model of lipid overload using a fatty acid mixture of oleic and palmitic acids in a 2:1 ratio (PA group), simulating conditions reminiscent of high-fat diet-induced hepatic steatosis. To assess the extent of lipid deposition within this model, we employed oil red and nile red staining. These analyses revealed a marked increase in lipid accumulation in the PA group (Figures 3A–C). In addition to these histological analyses, we examined the expression levels of genes related to lipid metabolism in cell model, including Acc1, Fasn, Cpt1, Scd1, Cd36, and Serbp1 mRNA. Our findings indicated an upregulation of Fasn, Acc1, Cpt1, Serbp1, and Cd36 in the PA group (Figure 3D).

### 3.5 Interplay between miR-200a-5p and AMPK pathway in PA cell model

**FIGURE 4:**
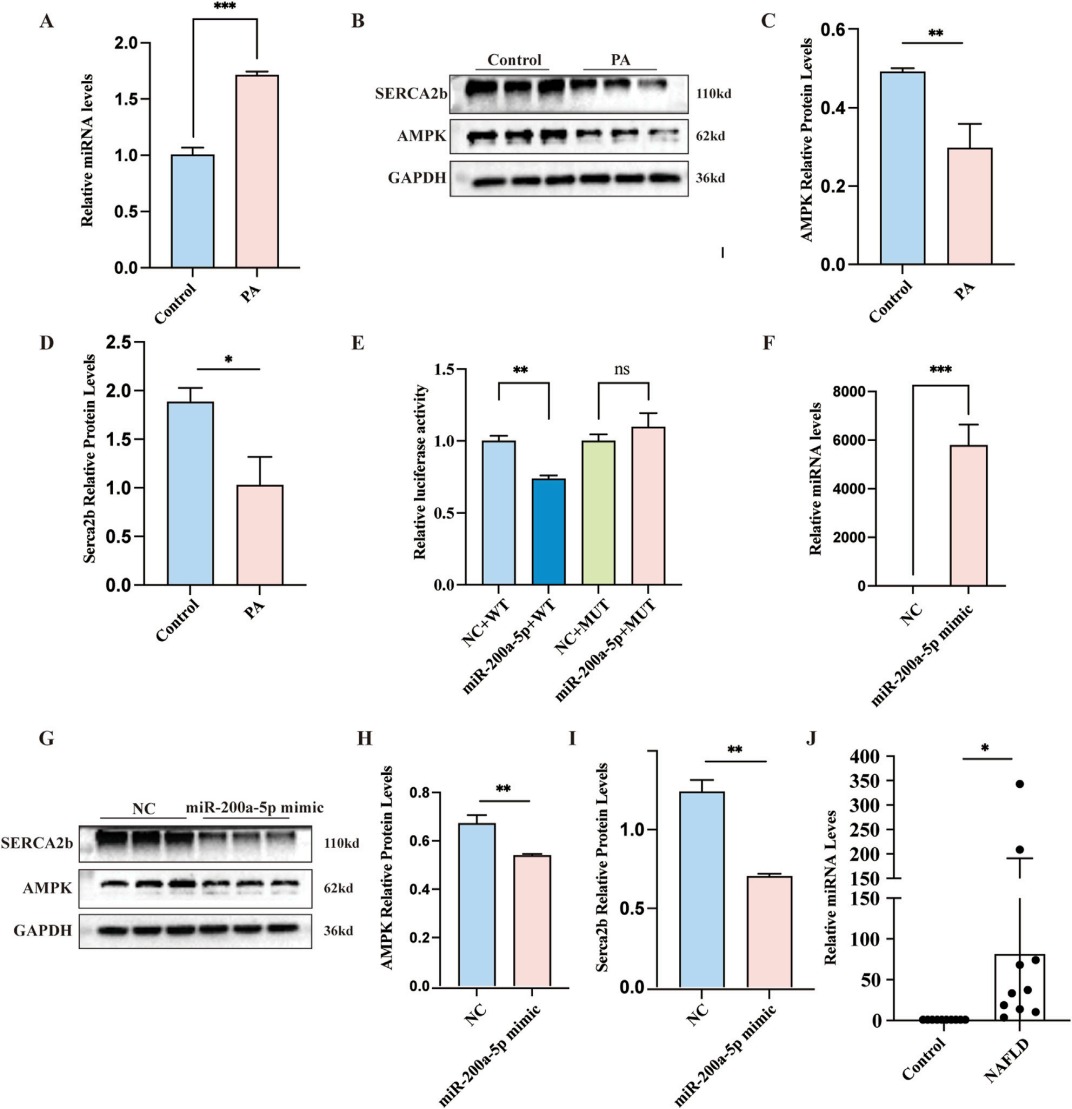
Exploring Lipid Metabolism and miRNA-200a-5p Regulation in Cells and patients. **(A)** Quantitative Real-Time PCR analysis showing miRNA-200a-5p expression in AML12 Cells. **(B–D)** Protein expression of AMPK and SERCA2b, as detected by Western blot, was normalized to GAPDH. **(E)** Luciferase reporter assay verified the relationship between miRNA-200a-5p and AMPK. **(F)** Quantitative Real-Time PCR analysis showing miRNA-200a-5p expression in AML12 Cells. **(G–I)** Protein expression of AMPK and SERCA2b, as detected by Western blot, was normalized to GAPDH. **(J)** Quantitative Real-Time PCR analysis showing miRNA-200a-5p expression in control individuals (n = 10) and MASLD patients (n = 10). Data were analyzed using one-way ANOVA. All values are expressed as mean ± SEM, with n = 3 per group. The groups are as follows: Control (normal group); PA (A mixture of Oleic and Palmitic Acids in a 2:1 ratio group); NC (normal group), miR-200a-5p mimic (miRNA-200a-5p mimic group). Comparisons were made between the PA group and the Control group, as well as between the miR-200a-5p mimic group and the NC group. Significance levels are indicated as follows: *p < 0.05, **p < 0.01, ***p < 0.001, ****p < 0.0001.

In our investigation of the PA cell model of hepatic steatosis, we observed a notable increase in miR-200a-5p expression within the PA group (Figure 4A). Concurrently, the expression levels of AMPK and SERCA2b elevated in the PA group as well (Figures 4B–D). To further explore the regulatory mechanisms, we employed a luciferase assay, revealing that the luciferase activity associated with the wild-type (WT) miR-200a-5p construct was lower than its negative control (NC) (Figure 4E). This finding suggests a direct interaction or regulatory effect mediated by miR-200a-5p. To validate the relationship between miR-200a-5p on the AMPK pathway, we overexpressed miR-200a-5p in the cells (Figure 4F). Subsequent analyses demonstrated that in the group treated with miR-200a-5p mimics, there was a reduction in the expression levels of AMPK and SERCA2b (Figures 4G–I).

### 3.6 The expression of miR-200a-5p serum levels in MASLD patients and healthy controls

To investigate the expression levels of miR-200a-5p in the peripheral serum of healthy individuals and MASLD patients, we collected serum samples from 10 healthy subjects and 10 MASLD patients. The basic demographic and biochemical profiles of these participants are detailed in Table 1. Using RT-PCR, we quantified the expression of miR-200a-5p in the serum samples of all 20 participants. The results revealed a significant elevation of miR-200a-5p levels in the serum of MASLD patients compared to the healthy controls. The difference in miR-200a-5p expression between the two groups was statistically significant (Figure 4J).

**TABLE 1:** Clinical patient information.

	N = 10	Control (mean ± SD)	N = 10	MASLD (mean ± SD)	P	
Height	10	162.50 ± 1.16	10	163.50 ± 2.70	0.74	NS
Weight	10	51.90 ± 2.06	10	74.70 ± 4.25	0.0001	***
BMI	10	19.81 ± 0.78	10	27.78 ± 0.92	<0.0001	****
Waist circ	10	70.70 ± 2.77	10	88.09 ± 3.06	0.0005	***
AST	10	17.43 ± 1.14	10	23.51 ± 1.59	0.01	**
ALT	10	14.57 ± 1.62	10	38.14 ± 6.93	0.0039	**
GGT	10	13.10 ± 1.24	10	96.50 ± 10.49	0.003	**
TC	10	3.91 ± 0.16	10	4.43 ± 0.31	0.16	NS
TG	10	0.88 ± 0.07	10	1.70 ± 0.31	0.02	*
HDL	10	1.53 ± 0.11	10	1.30 ± 0.08	0.12	NS
LDL	10	2.20 ± 0.16	10	2.67 ± 0.29	0.17	NS
GLU	10	4.78 ± 0.11	10	5.39 ± 0.16	0.01	**

Aspartate Aminotransferase (AST), Alanine Aminotransferase (ALT), Gamma-Glutamyl Transferase (GGT), Total Cholesterol (TC), Triglycerides (TG), High-Density Lipoprotein (HDL), Low-Density Lipoprotein (LDL), Glucose (GLU). Significance levels are indicated as follows: *P < 0.05, **P < 0.01, ***P < 0.001, ****P < 0.0001.

## 4 Discussion

**FIGURE 5:**
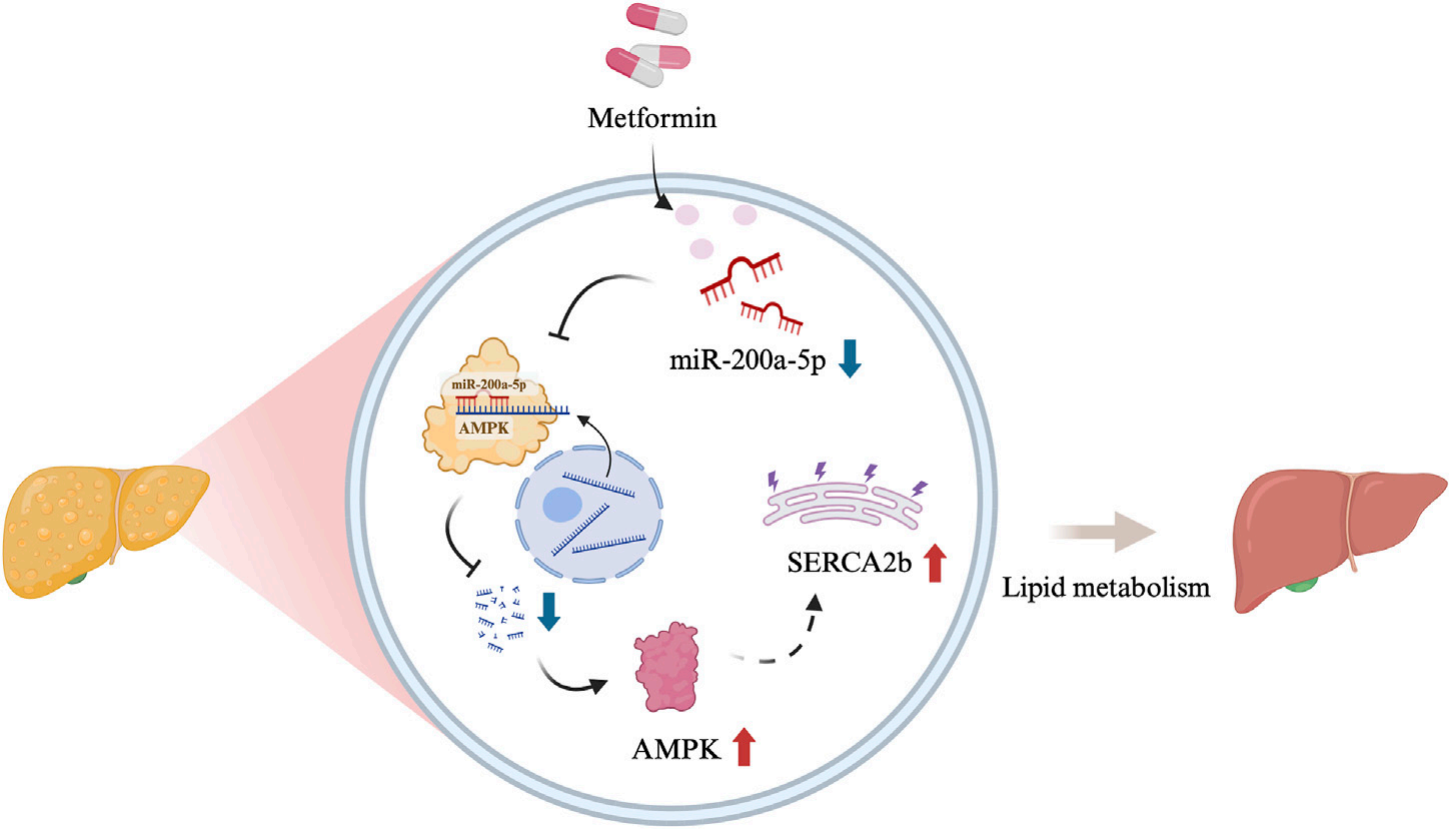
Graphical abstract [Created in BioRender. Chen, H. (2024)]. The schematic illustration showed the relationship among miR-200a-5p, AMPK and SERCA2b.

MASLD is characterized by dysregulated lipid metabolism and is frequently associated with obesity and metabolic syndrome. This study provides evidence that metformin can protect mice with MASLD induced by a high-fat diet by targeting the AMPK/SERCA2b pathway via miR-200a-5p. We observed that miR-200a-5p expression is elevated in MASLD, while metformin treatment significantly reduces its levels. These findings reveal a novel molecular mechanism by which metformin ameliorates MASLD and highlight the potential role of miRNAs in treating this condition (Figure 5).

Lipotoxicity is a key driver of MASLD, emphasizing the importance of lipid management. Dysregulated lipid metabolism elevates circulating free fatty acids, which surpass the storage capacity of adipose tissue and oxidative abilities of cells, causing ectopic lipid deposition and subsequent cellular damage. Free cholesterol has been shown to impair mitochondrial function, induce hepatocyte apoptosis, and accelerate MASLD progression (Cao and Kaufman, 2014; Welch et al., 2022). Moreover, in early steatosis, accumulated lipids, including cholesterol and triglycerides, compromise hepatocyte stability. This progression is further driven by lipid imbalance, mitochondrial dysfunction, ER stress, and oxidative stress, eventually advancing to NASH with fibrosis (Pang et al., 2018; Sanders and Griffin, 2016). Our study demonstrated that in both PA and HFD groups, there was an elevated expression of lipogenic genes (Fasn, Srebp1, Acc1, Scd1) and the fatty acid translocase Cd36, accompanied by significant increases in TG, TC, and LDL-C levels, and a decrease in HDL-C levels. Conversely, metformin treatment reduced the expression of these lipogenic genes and Cd36, correlating with decreased levels of TG, TC and LDL-C. These results, consistent with previous findings (Song et al., 2015; Hu et al., 2021), reaffirm that metformin can alleviate hepatic lipid metabolism disorders. Interestingly, while some studies have reported that metformin significantly elevates HDL-C levels (Feng et al., 2021; Paavilainen et al., 2022), our data did not show a notable increase in HDL-C after metformin treatment. One possible explanation for this discrepancy is the inherent variability of HDL-C levels, which may require a larger sample size to detect statistically significant changes between the HFD and HFD + Met groups.

MicroRNAs (miRNAs) play critical roles in various biological functions, including development, lifespan, cellular proliferation, differentiation, signaling pathways, apoptosis, and metabolism (Esquela-Kerscher and Slack, 2006). Previous research has shown that in patients with type 1 diabetes, metformin can downregulate miR-222, miR-195, and miR-21a, thereby protecting cardiac function and improving glycemic control (Ahmed et al., 2018). Further studies revealed that hepatocytes treated with lipotoxic palmitic acid exhibit elevated levels of miR-122 and miR-192, which correlate with the progression of steatohepatitis to liver fibrosis through upregulation of fibrosis-related genes such as smooth muscle actin (Li et al., 2017; Wang et al., 2009). Additionally, the choline-deficient L-amino acid-defined diet, known to induce steatohepatitis, has been shown to increase miR-122 and miR-192 levels in mice (Liu et al., 2018). Ezaz et al. recently reported significant elevations in miR-34a, miR-122, miR-192, and miR-200a expressions in a cohort of 132 patients with biopsy-confirmed MASLD, establishing a close histological association between miR-200a and MASLD severity (Ezaz et al., 2020). Furthermore, in rat livers with diet-induced MASLD, there was a significant downregulation of miR-122, miR-451, and miR-27, while miR-200a, miR-200b, and miR-429 were markedly upregulated (Alisi et al., 2011). The growing body of evidence suggests that circulating miRNAs are stable and detectable, making them potential candidates for monitoring MASLD severity and for non-invasive diagnosis. In our study, we focused on the variations in miR-200a-5p across different groups. Notably, we observed a differential expression of miR-200a-5p between the HFD and Metformin-treated groups. To further validate its function, we initially validated the interaction between miR-200a-5p and AMPK using a luciferase reporter assay in cell models. Subsequent overexpression of miR-200a-5p resulted in the reduction of AMPK levels.

Metformin is also known to alleviate ER stress. SERCA2b plays a crucial role in maintaining ER homeostasis, and reduced ER calcium levels due to decreased SERCA2b expression or activity can activate ER stress induced by obesity (Fu et al., 2011; Chen et al., 2021). Our previous studies found that miR-30b activates ER stress by targeting SERCA2b, leading to insulin resistance (Dai et al., 2019). Additionally, previous research has indicated that Maresin 1 ameliorates hepatic steatosis by inhibiting ER stress mediated by the AMPK/SERCA2b pathway (Jung et al., 2018). Our current findings suggest that in the treatment of MASLD with metformin, miR-200a-5p may modulate the expression of AMPK/SERCA2b, thereby ameliorating lipid deposition and ER stress. This provides new insights into the mechanisms through which metformin could effectively treat MASLD. However, our study has limitations, as we did not validate the miR-200a-5p and AMPK relationship at the animal level. Further investigation is needed to fully understand these mechanisms.

## 5 Conclusion

Our findings highlight miR-200a-5p as a potential target for reducing lipid deposition and ER stress via modulation of AMPK/SERCA2b, offering a new perspective on how metformin ameliorates MASLD. However, the lack of animal-level validation for the miR-200a-5p and AMPK interaction is a limitation of this study. Future research should address this gap to further elucidate the underlying mechanisms and confirm the therapeutic potential of this pathway.

## Data Availability

The original contributions presented in the study are included in the article/Supplementary Material, further inquiries can be directed to the corresponding authors.
