# Supplementary material for: Metformin’s effect on metabolic dysfunction-associated steatotic liver disease through the miR-200a-5p and AMPK/SERCA2b pathway
Source: Front Pharmacol. 2024 Dec 17;15:1477212. doi: 10.3389/fphar.2024.1477212 (PMC11685231; doi:10.3389/fphar.2024.1477212)
Supplement: Supplementary file 1 [file DataSheet1.docx]

Supplementary Material

# Supplementary Tables

**Supplementary Table 1.** The primers used for qRT-PCR assay.

| **Gene Name** | **Forward** | **Reverse** |
| --- | --- | --- |
| GAPDH | CAGTGGCAAAGTGGAGATTGTTG | TCGCTCCTGGAAGATGGTGAT |
| Serbp1 | TGGATTGCACATTTGAAGACAT | GCCAGAGAAGCAGAAGAG |
| Scd1 | CCGGAGACCCCTTAGATCGA | TAGCCTGTAAAAGATTTCTGCAAACC |
| Acc1 | GACGTTCGCCATAACCAAGT | CTGTTTAGCGTGGGGATGTT |
| Fasn | GCTGCGGAAACTTCAGGAAAT | AGAGACGTGTCACTCCTGGACTT |
| Cpt1 | TATTGGATGATGAAGCATATTACCG | ATAGTTAGTTGCCCACCATGACTT |
| Cd36 | GGAGTGCTGGATTAGTGGTTAG | GCTGTGAGCAGACGTATAGAAG |

# Supplementary Figures

# Supplementary Figure 1. Protein expression of AMPK activity by calculating the ratio of AMPKp/AMPK.


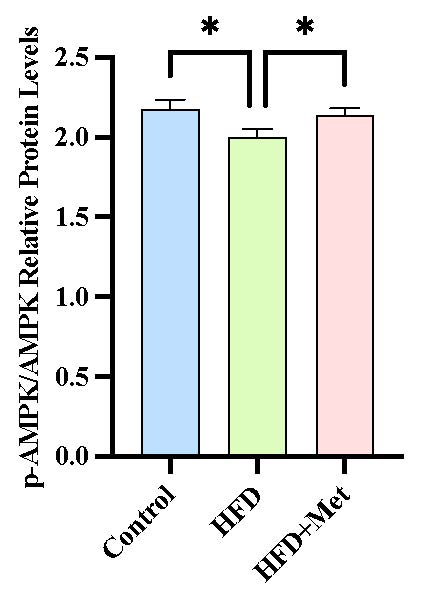


Protein expression of AMPK activity by calculating the ratio of AMPKp/AMPK. Data were analyzed using one-way ANOVA. All values are expressed as mean ± SEM, with n = 3 per group. The groups are as follows: Control (normal diet); HFD (high-fat diet); HFD + Met (mice on a high-fat diet receiving oral gavage of metformin at 300 mg/kg daily for the last 9 weeks). Comparisons were made between the HFD group and the Control group, as well as between the HFD + Met group and the HFD group. Significance levels are indicated as follows: *P < 0.05, **P < 0.01, ***P < 0.001, ****P < 0.0001.
